# Biochemical Adaptations of *Zea mays* L. to Abiotic Stress: A Focus on Drought, Salinity, and Heavy Metals

**DOI:** 10.1002/pei3.70217

**Published:** 2026-10-06

**Authors:** Sandra Etumah Ifie, Michael Ben Okon, Eliah Kwizera, Angela Mumbua Musyoka, Siida Robert, Vivian Onyinye Ojiakor, Augustine Oviosun, Chinyere Nneoma Ugwu, Conrad Ondieki Miruka, Josiah Eseoghene Ifie, Saidi Odoma, Abubaka Ibrahim Babangida, Patrick Maduabuchi Aja

**Affiliations:** ^1^ Department of Biochemistry, Faculty of Biomedical Sciences Kampala International University, Western Campus Ishaka Uganda; ^2^ Department of Anatomy, Faculty of Basic Medical Sciences, College of Medicine Enugu State University of Science and Technology Enugu Nigeria; ^3^ Department of Anatomy, Faculty of Biomedical Sciences Kampala International University, Western Campus Ishaka Uganda; ^4^ Department of Publication and Extension Kampala International University, Western Campus Kampala Uganda; ^5^ Department of Science Valley University of Science and Technology Bushenyi Uganda; ^6^ Department of Pharmacology Kampala International University Kampala Uganda; ^7^ Centre for Artificial Intelligence and Multidisciplinary Innovations Studies, Department of Auditing, College of Accounting Science University of South Africa Pretoria South Africa

**Keywords:** abiotic stress, antioxidant defense, climate resilience, CRISPR/Cas9, heavy metals, osmolytes, *Zea mays*

## Abstract

Maize ( *Zea mays* L.), one of the most significant cereal crops in the world, is very susceptible to abiotic stresses that include drought, salinity, and heavy metal toxicity, among others, all of which pose a risk to food and nutrition security in the face of climate change. These stresses disrupt cellular homeostasis, cause oxidative damage, inhibit photosynthesis, and eventually lower yield. This work is a synthesis of recent progress in knowledge of the biochemical, physiological, and molecular adaptation of maize to these conditions. The key processes involve reactive oxygen species (ROS) and hormone‐mediated signaling circuits; a strong antioxidant response (SOD, CAT, APX, and AsA‐GSH); osmolytes (proline, glycine, betaine, and soluble sugars); and metal detoxification (phytochelatin, metallothioneins, and transporter‐mediated) pathways. Genotypic variation can affect the effectiveness of these responses, and tolerant cultivars have been shown to have better redox regulation, ion homeostasis, and stress memory. Emerging biotechnological interventions, especially CRISPR/Cas9 genome editing and molecular breeding, offer promising opportunities to accelerate the development of multi‐stress‐tolerant maize. Nevertheless, there are knowledge gaps in the understanding of combined stress responses, the role of epigenetic regulation, and the field‐level translation of biochemical processes into yield stability. This review highlights the necessity to adopt integrative and systems‐level solutions that can integrate the understanding of the molecular basis, breeding, and biotechnology to improve the resilience of maize, protect productivity, and sustain agriculture in light of increasing climate variability.

## 1 Introduction

Climate change is on the rise, and it is causing complex changes in the global ecosystems and agriculture. Changing the soil–plant‐atmosphere continuum, which directly depends on crop productivity and food security, is one of the most significant effects (Oshunsanya et al. 2019). Even though world food production has been able to keep up with or even exceed demand over the last few decades (Vermeulen et al. 2010), this equilibrium is still delicate, especially in developing countries. Agriculture is still the livelihood activity of almost 70% of the population in Africa, Asia, and the Asia‐Pacific, with much of the population living in arid and semi‐arid environments where severe weather conditions challenge food production (Yadav et al. 2018, 2018). Plants are sessile, unlike mobile organisms, and they cannot avoid unfavorable environments. Rather, they depend on inherent physiological and biochemical processes to withstand the stress. Nonliving environmental factors like drought, salinity, flooding, extreme temperatures, nutrient deficiencies, and contamination of metals are abiotic stresses that disrupt growth, metabolism, and the potential yield (Gull et al. 2019; Langangmeilu et al. 2024). They are especially devastating since they affect several plant life cycles, including germination and vegetative growth, reproductive, and grain filling (Rahim et al. 2025). Moreover, it is common to observe that in many cases, combined stresses, e.g., drought and heat/salinity, have more detrimental effects than single stresses do (Kumar 2020). The abiotic stress impacts plants in various ways, such as osmotic and oxidative stress, cellular damage, disturbed nutrient homeostasis, interrupted photosynthetic pigments and enzymatic processes, and protein denaturation (Chakraborty et al. 2021; Reddy et al. 2024). The stressors weaken the plant's control over its water balance, ion uptake, and cell integrity. These stresses present a major challenge at the ecosystem level to the resilience of agriculture and long‐term food security.

Maize ( *Zea mays* L.), which is one of the three most important cereal crops in the world after wheat and rice, is very susceptible to these changes. Maize, also known as the “queen of cereals” because of its significance in the world, was domesticated more than 9000 years ago and is currently at the centre of global food, feed, and industrial systems (Tanumihardjo and Nuss 2010; Grote et al. 2021; Erenstein et al. 2022). Maize in sub‐Saharan Africa accounts for almost half of the total cereal production, with over 80% of production being used as food, feed, and industrial (FAOSTAT 2016). Maize is also the source of essential raw materials of starch, oil, alcoholic beverages, sweeteners, and biofuels (Dragomir et al. 2022). In spite of this flexibility, its vulnerability to environmental changes poses a threat to the regional food and the entire global food system. An example of maize susceptibility is drought stress. Water scarcity during sensitive developmental stages, like silking, causes a severe reduction in the number of kernels set, grain fill, and yield (Tang et al. 2025). On the cellular level, drought impairs photosynthesis, decreases the canopy leaf area, prolongs the anthesis‐silking interval (ASI), and decreases grain weight (Almeida et al. 2014; Salgado‐Aguilar et al. 2020). In the worst‐case scenario, drought may completely stop the harvest, which threatens the food security of households (Jing et al. 2023; Danish et al. 2024). In the same way, salinity pressure has become a formidable environmental limitation to the production of maize. The formation of salt in soils decreases the amount of water and creates an ionic imbalance, especially of sodium ions (Na^+^), which leads to cellular toxicity and inhibits growth (Fortmeier and Schubert 1995; el Sabagh et al. 2024). Maize is also very sensitive to salinity in its early growth, where seed germination and seedling development are usually impaired in salty environments (Billah et al. 2017; Feng et al. 2017). This stress ultimately decreases yields and affects agricultural sustainability in the concerned areas.

Another serious abiotic stress is heavy metal contamination, especially in agricultural systems that are close to industrial areas or subjected to polluted irrigation water. Cadmium (Cd), lead (Pb), and mercury (Hg) are metals that accumulate in soils, inhibit the germination and growth of seeds, and decrease yields (Chen et al. 2018; Wang et al. 2021; Atta et al. 2023). More than lowering productivity, such metals bioaccumulate in various edible plant tissues, posing a risk to food safety and human health (Zhao et al. 2014; Yang et al. 2022). Negative effects of toxic metals (Pb and Cr) are the inhibition of photosynthesis, decrease in biomass, and visible symptoms, including chlorosis and retarded growth (Rai et al. 2019; Vasilachi et al. 2023). Ecological persistence makes the issue even worse because metals stay in the soil over a long time and still represent a threat to crops and consumers. In spite of these obstacles, plants have developed several methods of adaptation to reduce abiotic stress. These are morphological adaptations (e.g., a change in root architecture), physiological adaptations (e.g., stomatal control and osmoprotectant levels), and molecular adaptations (e.g., regulation of genes, reactive oxygen species (ROS) neutralization, and hormonal signaling via abscisic acid, ethylene, salicylic acid, and jasmonic acid) (Devi et al. 2018; Taria et al. 2022; Zhang et al. 2023). Plants are also prepared to face repeated stresses with epigenetic memory, transcriptional reprogramming, and systemic acquired resistance (SAR) (Hasanuzzaman and Fujita 2023). In the case of maize, genetic enhancement of such mechanisms and molecular breeding, as well as biotechnological developments, can provide promising solutions to resilience. Together, drought, salinity, and heavy metal stress are some of the most important abiotic limitations on maize productivity in the world. Not only do they pose a risk to yields and crop quality, but they also put global food and nutrition security at risk. This review thus aims at giving a detailed insight into the process of maize resistance to these stresses, with special reference to biochemical reactions and current developments in the study of plant adaptation. The review synthesizes existing information to outline approaches to enhance the resilience of maize and to guarantee the sustainability of agriculture when confronted by climate change.

## 2 Stress Signaling and Regulatory Pathways

Drought, salinity, and heavy metal toxicity are all abiotic stresses that severely affect the productivity of maize ( *Zea mays* L.) by disrupting the homeostasis of the cellular system and causing oxidative and ionic imbalances (Shafiq et al. 2022). Maize depends on reactive oxygen species (ROS)‐mediated and phytohormonal‐controlled highly coordinated stress signaling networks to survive such conditions, which locally and systemically trigger adaptive responses (Mohanta et al. 2018). These pathways are not independent of each other, but instead they interact in a wide‐ranging crosstalk that allows plants to adapt responses to different stress types, intensities, and durations.

### 2.1 ROS Signaling as a Central Mediator

The production of ROS, such as superoxide radicals (O_2_^−^), hydrogen peroxide (H_2_O_2_), hydroxyl radicals (OH), and singlet oxygen (O_2_), is a continuous process in the aerobic metabolism of maize that takes place in organelles, which include chloroplasts, mitochondria, and peroxisomes (Sharma et al. 2012). ROS controls growth and developmental processes under favorable conditions, but abiotic stresses increase its generation significantly, causing oxidative stress (Gill and Tuteja 2010). It is the duality of ROS that plays a central role in stress physiology: whereas an uncontrolled accumulation of this molecule causes lipid, protein, and nucleic acid damage, a regulated burst of ROS acts as an intermediary in signaling and triggers the activation of defense mechanisms (Sies et al. 2022). Stomata are closed during drought, which reduces the entry of CO_2_, thereby excessively blocking the photosynthetic electron transport chain and increasing the production of ROS in chloroplasts (Yang et al. 2021). Salinity interferes with ionic balance, and heavy metals (cadmium Cd and lead Pb) further enhance the accumulation of ROS by interrupting the electron transport and triggering Fenton reactions leading to the production of hydroxyl radicals (Shahid et al. 2020). Plasma membrane NADPH oxidases (respiratory burst oxidase homologs, RBOHs) are significant sources of ROS during stress, which convert NADPH‐derived electrons to superoxide, which in turn is converted to H_2_O_2_, a relatively stable molecule that diffuses through aquaporins to serve as a systemic signal (Mittler et al. 2022; Zhao et al. 2023). ROS signaling can affect various downstream events, especially via mitogen‐activated protein kinase (MAPK) cascades, which activate transcription factors like DREB, NAC, and WRKY that control stress‐regulated genes like osmolyte synthesis, antioxidant defense, and ion homeostasis (Arbona et al. 2013; Zhao et al. 2023). Moreover, ROS also responds to calcium (Ca^2+^) signaling, forming positive feedback where Ca^2+^ activates RBOHs, increasing ROS production, and Ca^2+^ increases defense activation with ROS, thereby providing strong and quick defense activation (Ravi et al. 2023). Taken together, these highly controlled ROS processes constitute the basis of maize abiotic stress sensing and response.

### 2.2 Hormonal Control of Stress Responses

Along with ROS signaling, phytohormones, such as abscisic acid (ABA), ethylene (ET), and salicylic acid (SA), are systemic messengers that coordinate cellular and physiological responses. These hormones hardly work alone but rather integrate with ROS signals to organize the regulation of stomata, antioxidant defense, and metabolic reorganization (Arbona et al. 2013; Nambara and van Wees 2021). ABA is the main controller of the maize reactions to drought and salinity. It is quickly upregulated in osmotic stress through its biosynthesis, which is regulated by NCED enzymes (Dar et al. 2017). ABA signaling facilitates stomatal closing through the ABA‐ROS‐Ca^2+^ cascade, in which NADPH oxidase‐produced H_2_O_2_ serves as a secondary messenger to open ion channels in guard cells and minimize water loss (Wani et al. 2016; Yang et al. 2021). In addition to stomatal regulation, the ABA‐SnRK2 signaling pathways coordinate stress‐responsive transcription factors, promoting osmoprotectants, LEA proteins, and antioxidant enzyme accumulation (Liu et al. 2024). ABA reinforces root barrier development and restricts the movement of toxic ions to aerial tissues under heavy metal stress, which results in tolerance. Ethylene, commonly known as a stress hormone, is a fine‐tuned regulator of stress in maize. Whereas the high level of ethylene may induce growth deterrence and early senescence, moderate levels enhance adaptive phenotypes, such as adventitious root development, modified shoot elongation, and enhanced antioxidant activity (Shi et al. 2017; Khan, Alvi, et al. 2024). Ethylene has a strong relationship with ROS: it may trigger the production of ROS to signal but at the same time increases the expression of antioxidant enzymes to prevent oxidative damage (Raja et al. 2017). It has been observed that manipulation of ethylene sensitivity, revealed in ZmARGOS8‐overexpressing maize, enhances the drought resistance and the yield (Shi et al. 2017). Although salicylic acid (SA) is traditionally linked to pathogen defense, it also plays a role in the adaptation of maize to abiotic stress. SA increases the activity of antioxidant enzymes, suppresses the toxicity of ROS, and preserves photosynthetic rates in the case of a long‐term drought and salinity (Khan et al. 2022). It also controls ionic balance during salinity stress by promoting Na^+^ exclusion and K^+^ retention, thereby facilitating the maintenance of cellular ion homeostasis (Jayakannan et al. 2015; Mohammadi et al. 2022). SA enhances the process of detoxification in heavy metal stress by inducing glutathione and phytochelatin production, which traps the toxic ions in vacuoles (Gunes et al. 2019). SA acts in a complex crosstalk with ABA and antagonistically with ABA in stomatal control and synergistically with ethylene to enhance antioxidant defenses (Singh and Roychoudhury 2023).

### 2.3 Integrated Stress Response Networks

Maize evidence shows that this network cannot be reduced to a uniformly protective ABA‐ROS response. ZmbZIP4 and the ZmbHLH47‐ZmSnRK2.9 module promote drought resistance through ABA‐associated signaling and root or stomatal responses, whereas MAPK‐like protein 1 increases drought sensitivity by suppressing ABA biosynthesis. Likewise, ZmAL14 phosphorylation by ZmSnRK2.2 promotes drought resistance, but ZmGCT1/2 oppose stomatal closure by inhibiting ZmSLAC1 (Ma et al. 2018; Zhu et al. 2020; Yan et al. 2024; Wang, Cheng, et al. 2024; Feng et al. 2025). These apparently divergent effects indicate node‐, tissue‐, and developmental‐stage specificity rather than a simple positive relationship between ABA abundance and tolerance. The central unresolved issue is which combinations preserve carbon gain and reproductive development while limiting water loss under field conditions.

## 3 Antioxidant Defense Mechanisms in *Zea mays* L. Under Abiotic Stress

Section 2 established ROS as both a signal and a source of injury. This section therefore focuses only on the downstream maize antioxidant machinery and its evidential limits. In maize, stress‐induced superoxide and hydrogen peroxide are processed by compartment‐specific enzymatic systems and the ascorbate‐glutathione cycle; however, higher antioxidant activity may reflect greater oxidative injury as well as greater protective capacity. Causal evidence and yield‐level validation are therefore more informative than activity measurements alone (AbdElgawad et al. 2016; Muhammad et al. 2023; AbdElgawad et al. 2020).

Superoxide dismutase (SOD) converts superoxide to H_2_O_2_, after which catalase (CAT) and ascorbate peroxidase (APX) remove H_2_O_2_ in different compartments. Maize roots and shoots do not respond identically: their antioxidant profiles diverge under salinity and heavy‐metal exposure, so whole‐seedling averages can obscure the organ in which protection occurs (AbdElgawad et al. 2016; AbdElgawad et al. 2020). Melatonin priming increases antioxidant capacity and drought performance in seedlings, but this treatment evidence does not establish that constitutively high enzyme activity will improve grain yield across environments (Muhammad et al. 2023).

The non‐enzymatic arm is coupled to APX through ascorbate (AsA), glutathione (GSH), dehydroascorbate reductase, and glutathione reductase. Redox ratios such as AsA/DHA and GSH/GSSG are more informative than a single enzyme measurement because they report recycling capacity. Nevertheless, genotype, tissue, stress dose, and sampling time strongly influence these ratios; they should be interpreted with membrane damage, photosynthesis, recovery, and yield rather than used as stand‐alone tolerance markers (Rizk et al. 2024; Eskikoy and Kutlu 2024).

Maize‐specific genetic studies further show that redox control is embedded in broader regulatory networks. Co‐expression of ZmVPP1 and ZmNAC111 improves drought resistance, while ZmSRG7 affects both drought and salt responses, linking antioxidant protection with proton transport and transcriptional regulation (Liu et al. 2022; Wei et al. 2022). The practical question is not whether antioxidants are induced, but whether manipulating a defined node improves reproductive‐stage performance without penalizing growth under non‐stress conditions.

Accordingly, antioxidant defenses are treated here as one interacting component of maize acclimation, not as an independent mechanism repeated in subsequent sections. Osmolyte‐redox coupling is discussed in Section 4, metal‐associated redox responses in Section 5, and whole‐plant consequences in Section 6. Figure 1 summarizes the maize‐centered signaling‐to‐detoxification sequence, whereas Figure 2 focuses on metabolite coupling.

**FIGURE 1:**
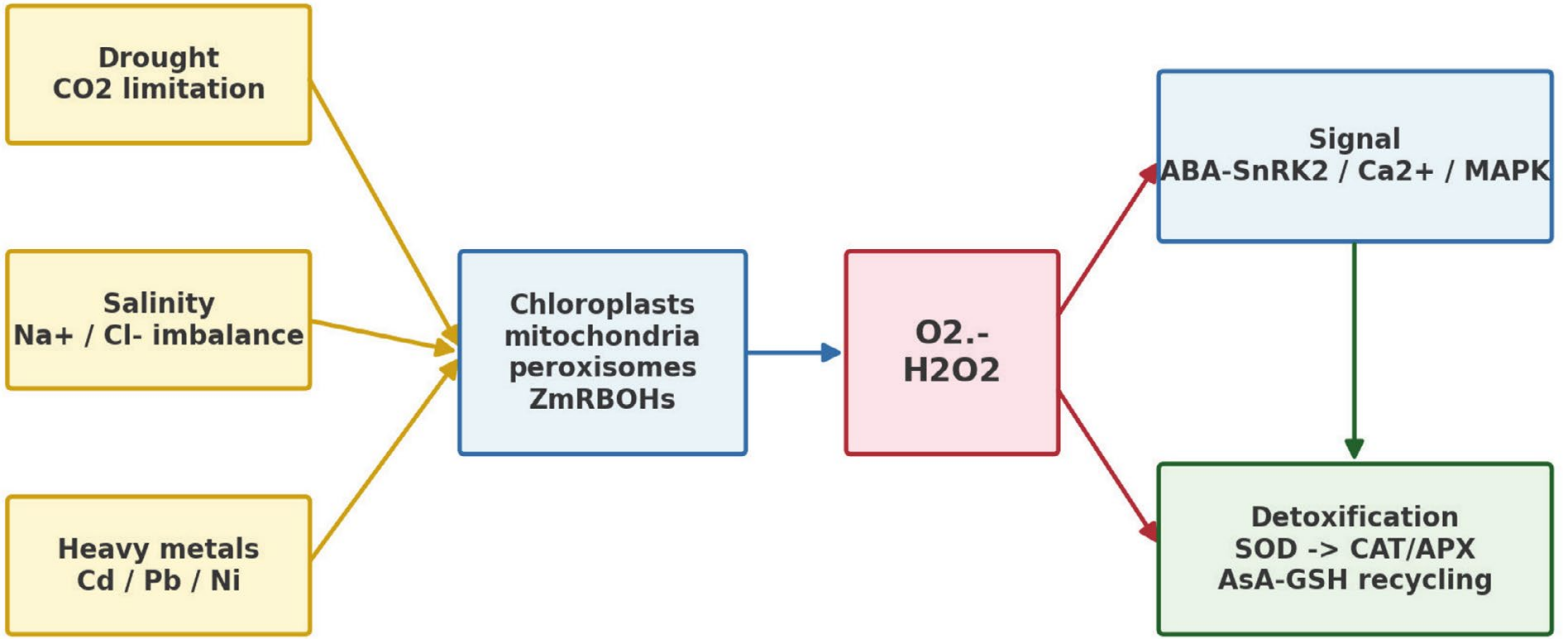
Maize ROS signaling and antioxidant control.

**FIGURE 2:**
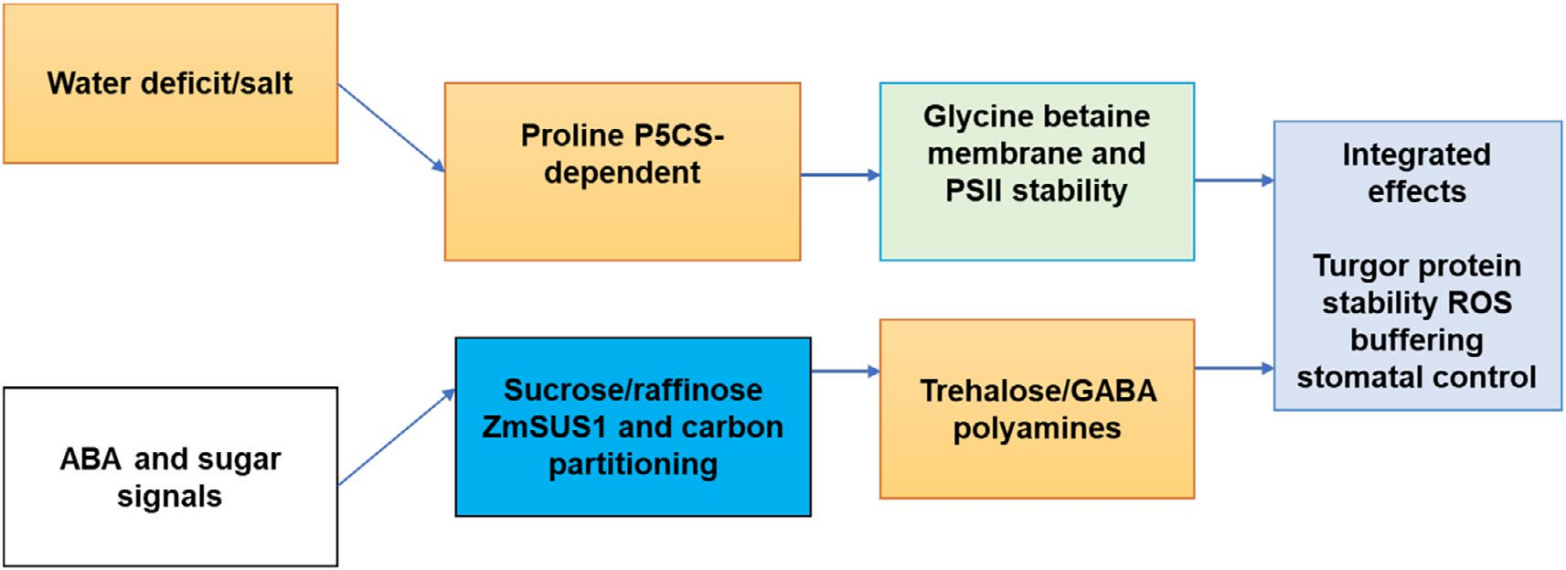
Maize osmolyte metabolism coupling to water, carbon, and redox status.

## 4 Osmolyte Accumulation in *Zea mays* Under Abiotic Stress

Compatible solutes in maize connect osmotic adjustment with carbon allocation, membrane protection, and redox buffering. Their contribution varies with genotype, organ, developmental stage, and stress history; therefore, concentration alone is not a sufficient tolerance criterion. Maize evidence for raffinose, sucrose metabolism, and genotype‐dependent salt responses provides a stronger basis than extrapolation from other plant species (Liu, Li, et al. 2023; Xiao et al. 2024; Rizk et al. 2024).

Proline commonly accumulates in drought‐, salt‐, and metal‐exposed maize and can support osmotic adjustment, protein stability, and redox buffering. Maize studies report stress‐associated increases in proline, but the direction and magnitude depend on genotype and treatment (Pingle et al. 2022; Tiwari 2024). Because accumulation can also indicate severe stress, future work should distinguish synthesis from reduced catabolism and relate proline flux to recovery and grain yield.

Glycine betaine can stabilize proteins and photosynthetic membranes under dehydration and salinity. In maize, evidence includes altered glycine‐betaine biosynthesis and improved Na^+^ homeostasis after exogenous application or pathway manipulation (Phillips et al. 2018; Zhu, Li, et al. 2022). However, treatment studies and transgenic expression should be compared with endogenous genotype variation and reproductive‐stage yield before glycine betaine is used as a breeding proxy.

Soluble sugars are both osmotic compounds and metabolic signals. Maize‐specific evidence links ZmSUS1‐driven sucrose metabolism and raffinose accumulation with drought resistance and reduced transpiration (Liu, Li, et al. 2023; Xiao et al. 2024). The benefit is context dependent because diverting carbon into storage or compatible solutes can protect cells while constraining growth; measurements of carbon flux and kernel set are needed to resolve this trade‐off.

Additional protective layers are provided by other osmolytes like trehalose, γ‐aminobutyric acid (GABA), and polyamines. Trehalose protects the proteins and ensures osmotic balance, whereas GABA helps stabilize pH and signaling (Li, Cheng, et al. 2020; Dubey et al. 2021). Polyamines such as putrescine and spermidine increase in response to heavy metals and decrease oxidative damage and lipid peroxidation in most cases in concert with proline and sugars (Hasanuzzaman et al. 2019; Piao et al. 2022). This concerted action of these metabolites indicates the combined reprogramming of the maize metabolism in response to stress.

Osmolyte accumulation is coupled to ABA signaling, carbon allocation, antioxidant recycling, and tissue transport rather than functioning as an isolated endpoint. Maize studies provide causal support for particular components: raffinose reduces leaf transpiration, and ZmSUS1‐mediated sucrose metabolism increases soluble sugar content and drought resistance (Liu, Li, et al. 2023; Xiao et al. 2024). Even so, a high proline or sugar concentration can mark injury‐driven metabolic arrest rather than effective acclimation. Future comparisons should quantify synthesis and turnover fluxes, tissue localization, recovery, and yield, and should test whether osmotic benefits offset the carbon cost of solute production.

## 5 Metal Detoxification Mechanisms in *Zea mays*

Maize is exposed to essential metals at excessive concentrations and to non‐essential toxic metals through contaminated soils, irrigation water, and amendments. The agronomic problem includes growth inhibition and food‐chain transfer, so studies should evaluate both plant tolerance and grain safety. Direct maize experiments demonstrate metal‐specific, organ‐specific, and genotype‐dependent responses rather than one universal detoxification program (Atta et al. 2023; AbdElgawad et al. 2020; Zamani et al. 2024).

Metal exposure initiates Ca^2+^ and ROS signals and alters transcription, but direct functional evidence for individual maize MAPKs and transport regulators remains uneven. The strongest maize data show organ‐specific oxidative and antioxidant profiles under metal toxicity and genotype‐dependent responses to combined salinity and Pb stress (AbdElgawad et al. 2020; Zamani et al. 2024). These results caution against transferring pathway assignments from Arabidopsis, rice, or papaya to maize solely on the basis of gene‐family homology. Figure 3 therefore distinguishes established response classes from transporter and regulatory steps that still require maize loss‐of‐function validation.

**FIGURE 3:**
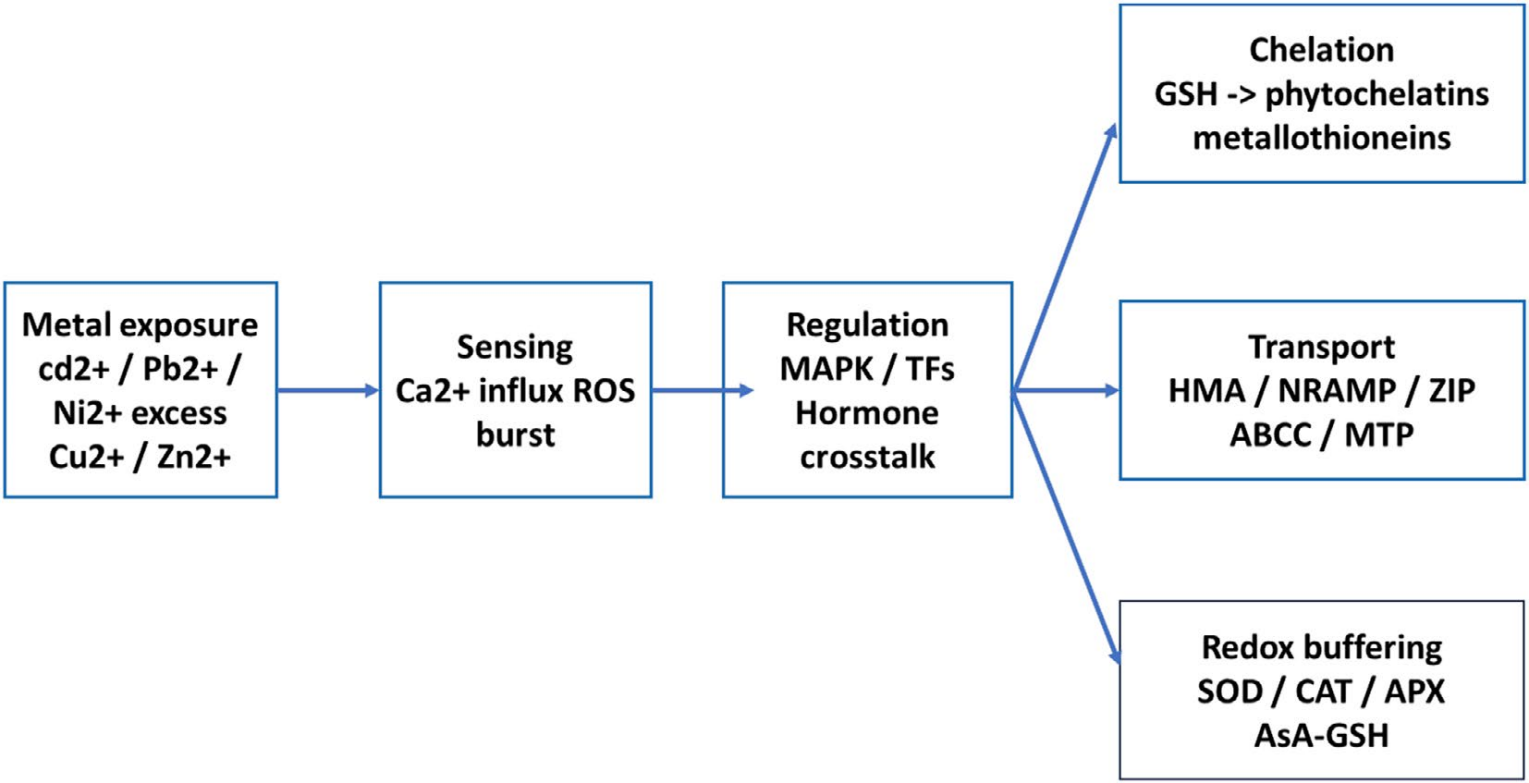
Maize heavy‐metal response from sensing to detoxification.

Chelation by glutathione‐derived phytochelatins, metallothioneins, and organic acids can reduce the free‐metal pool, while membrane transport and vacuolar sequestration limit cytosolic exposure. In maize roots exposed to Ni, silicon treatment was associated with chelation, detoxification, and altered membrane transport, but treatment‐mediated mitigation does not by itself identify the responsible transporter (Lakhneko et al. 2024). Claims for specific ZmABCC, ZmHMA, ZmNRAMP, or ZmMTP proteins should therefore be separated into experimentally validated functions and homology‐based candidates.

Sequestration can protect maize by immobilizing metals in root cell walls or vacuoles and restricting movement to photosynthetic and reproductive tissues (Figure 4). This strategy may improve crop tolerance and grain safety but can reduce shoot accumulation, which is undesirable when phytoextraction is the objective. Reporting only biomass without tissue metal concentrations cannot distinguish these outcomes (AbdElgawad et al. 2020; Zamani et al. 2024).

**FIGURE 4:**
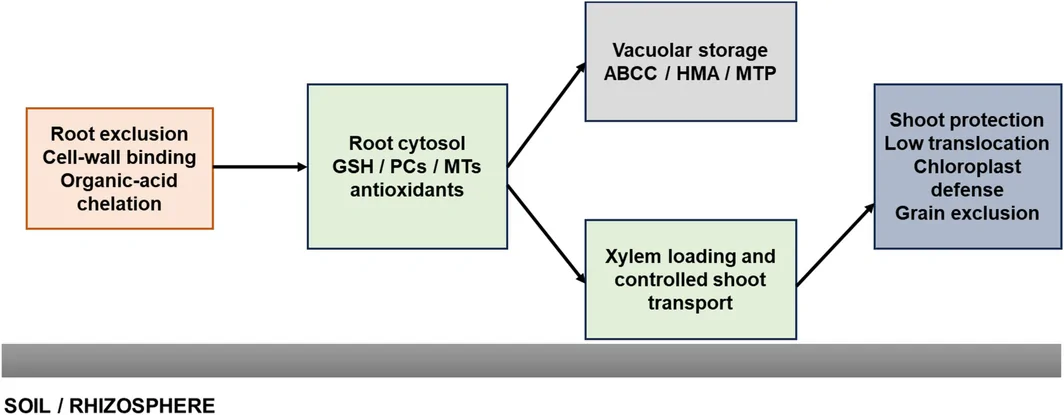
Root‐to‐shoot metal handling in maize.

Metal‐generated ROS are controlled by the antioxidant network described in Section 3; repeating its enzyme chemistry here would obscure the metal‐specific questions. In maize, the more discriminating endpoints are root‐versus‐shoot redox status, metal speciation, subcellular localization, xylem loading, and grain translocation (AbdElgawad et al. 2020; Zamani et al. 2024). These endpoints also separate crop protection, which favors root retention and grain exclusion, from phytoextraction, which requires substantial shoot accumulation.

Transport proteins can mediate both nutrient acquisition and toxic‐metal movement, making selectivity and tissue localization critical. Candidate ZIP, NRAMP, HMA, ABCC, and MTP proteins should not be assigned maize functions from homology alone; loss‐of‐function, complementation, subcellular localization, and metal‐speciation experiments are needed. Current maize treatment and genotype studies support altered transport as a response class but leave several individual assignments unresolved (Lakhneko et al. 2024; Zamani et al. 2024).

Hormone and ROS crosstalk probably coordinates metal responses with growth, but this evidence is less gene‐specific in maize than for drought and salinity. Future studies should test whether ABA, ethylene, salicylic acid, or jasmonate perturbations change metal speciation and grain transfer rather than relying only on antioxidant activity. This distinction prevents general plant hormone models from being presented as established maize detoxification mechanisms.

## 6 Physiological Outcomes and Stress Tolerance in *Zea mays* Under Abiotic Stress

The physiological outcome of stress in maize emerges from water status, ion balance, carbon assimilation, redox control, and reproductive timing rather than from any single biochemical marker. Responses vary among genotypes and organs: comparative maize studies show diversity in drought‐associated biochemical traits, salt‐associated ionomes and antioxidant systems, and salinity‐Pb interactions (Eskikoy and Kutlu 2024; Khan, Gao, et al. 2024; Zamani et al. 2024). Table 1 summarizes outcomes, while the mechanisms themselves are cross‐referenced to Sections 2, 5 to avoid repetition.

**TABLE 1:** Physiological outcomes of abiotic stresses in maize (*Zea mays L*.).

Stress type	Growth effects	Photosynthesis effects	Yield outcomes	Reported yield loss (%)	References
Drought	Reduced leaf area, plant height, biomass	Lower CO_2_ assimilation, chlorophyll loss, PSII damage	Reduced grain yield, kernel number, harvest index	Up to 50%; 3%–8% loss per day at flowering	Kim and Lee (2023); Gill and Tuteja (2010)
Salinity	Reduced seedling vigor, shoot/root growth	Chlorophyll degradation, ROS damage, impaired electron transport	Poor grain set, reduced kernel weight, germination issues	30%–60% depending on severity	Iqbal et al. (2020); Wang, Chen, and Sui (2024)
Heavy metals (Cd, Pb, Cu)	Stunted shoot/root growth, necrosis, nutrient imbalance	Reduced chlorophyll, thylakoid destabilization, impaired PSII	Poor grain filling, reduced ear size, seed development	Variable; Cd up to 40%–60%	Shafiq et al. (2022); Rashid et al. (2023)
Combined stress (drought + salinity/heat/metal)	Altered root morphology, reduced biomass	Synergistic oxidative damage, impaired photosynthesis	Severe yield losses, reduced harvest index	> 50%–65% under combined drought + Cd	Kavian et al. (2023); Fan et al. (2024)

Drought effects depend on intensity, duration, developmental stage, root class, and genotype. Maize axial position and root class alter drought responses, and large root cortical cells can reduce the metabolic cost of soil exploration (Hazman and Kabil 2022; Chimungu et al. 2014). At the leaf level, stomatal closure conserves water but restricts CO_2_ assimilation; raffinose‐mediated reduction of transpiration and ABA‐linked guard‐cell modules therefore present a trade‐off between water saving and carbon gain (Liu, Li, et al. 2023; Yan et al. 2024; Feng et al. 2025). Because kernel set is especially sensitive around flowering, seedling survival should not be treated as a proxy for yield stability.

Salinity combines an early osmotic phase with later Na^+^ and Cl^−^ toxicity. Maize‐specific evidence identifies natural genetic variation, a major field‐stage QTL, the ZmbHLH32‐ZmIAA9‐ZmARF1 module, and ZmEREB57‐dependent oxylipin signaling as contributors to tolerance (Sandhu et al. 2020; Luo et al. 2017, 2021; Yan et al. 2023; Zhu et al. 2023). These studies also expose a scale problem: seedling biomass, ion ratios, and antioxidant activity do not always predict mature‐plant yield, and acclimation history can change the apparent ranking of genotypes (Pandolfi et al. 2016; Singh et al. 2023).

Heavy‐metal effects in maize depend on metal identity, chemical availability, dose, exposure route, and genotype. Roots and shoots show distinct oxidative profiles, and tolerant and sensitive genotypes differ under combined salinity and Pb stress (AbdElgawad et al. 2020; Zamani et al. 2024). These data support organ‐resolved analyses but do not justify assuming that greater shoot accumulation is agronomically desirable: tolerance, grain safety, and phytoextraction are different objectives. Field studies should therefore report soil chemistry, root retention, shoot transfer, and grain concentrations together with biomass and yield.

Cross‐study comparison remains limited by different genotypes, growth stages, stress intensities, and endpoint definitions. Combined stresses are not reliably additive: maize exposed to drought plus cold undergoes coordinated transcriptional and metabolomic adjustment that cannot be inferred from either single stress alone (Guo et al. 2021). A tolerance claim is strongest when biochemical change is linked to recovery, reproductive performance, and multi‐environment yield; correlations from a single tissue or time point should be framed as candidate mechanisms.

## 7 Biotechnological Interventions

Biotechnology can accelerate maize improvement, but the evidential bar should be trait performance across developmental stages and environments, not molecular change alone. Useful strategies include gene editing, transgenic stacking, marker‐assisted selection, genomic prediction, and microbiome‐assisted approaches. Their comparative value depends on effect size, pleiotropy, genetic background, regulatory feasibility, and whether tolerance persists under combined stresses.

### 7.1 Genetic Engineering

Maize engineering has progressed from transgenic stress‐response genes to targeted editing and pathway stacking. Editing ZmGA20ox3 improved plant architecture and drought tolerance, whereas co‐expression of ZmVPP1 and ZmNAC111 produced stronger drought resistance than single‐pathway reasoning would predict (Liu, Chen, et al. 2023; Liu et al. 2022). Field evidence remains comparatively scarce: altered ethylene biosynthesis has increased maize grain yield under field drought, illustrating the importance of reproductive‐stage validation (Habben et al. 2014). Candidate genes with positive seedling phenotypes may still impose growth or yield penalties; multi‐location testing and near‐isogenic comparisons are therefore required before deployment.

### 7.2 Molecular Breeding

Molecular breeding should prioritize loci that reproduce across genetic backgrounds and field environments. Maize GWAS and natural‐variation studies have identified salt‐tolerance candidates, while a major QTL has been mapped in mature field‐grown plants (Sandhu et al. 2020; Luo et al. 2017, 2021). Genome‐wide prediction can capture polygenic biomass responses, but prediction accuracy and marker effects are population‐ and environment‐dependent (Singh et al. 2023). For drought, genotype comparisons and root phenotyping show that physiological architecture must accompany marker data (Eskikoy and Kutlu 2024; Hazman and Kabil 2022). The key translational gap is validation of favorable alleles in elite germplasm under drought, salinity, heavy metals, and their combinations.

## 8 Critical Appraisal: Conflicting Evidence, Limitations, and Unresolved Questions

Maize regulatory studies reveal both positive and negative drought controllers. ZmEREBP60 and ZmbHLH47‐ZmSnRK2.9 enhance tolerance, whereas ZmASR1, ZmBES1/BZR1‐1, MAPK‐like protein 1, and ZmGCT1/2 reduce tolerance through distinct effects on ABA, stomata, or downstream transcription (Zhu, Liu, et al. 2022; Yang et al. 2024; Feng et al. 2023, 2025; Zhu et al. 2020; Yan et al. 2024). These findings are not interchangeable and show why generalized statements that ABA or a transcription‐factor family ‘promotes tolerance’ are insufficient.

The literature is dominated by controlled‐environment and seedling experiments, and some maize genes are evaluated only in heterologous species. Such studies establish biological plausibility but cannot demonstrate maize yield stability. Field evidence, including ethylene‐pathway manipulation and mature‐plant salinity QTL mapping, is more directly translational but remains uncommon (Habben et al. 2014; Luo et al. 2017). Genotype‐by‐environment interaction, reproductive‐stage sensitivity, and growth‐defense trade‐offs should therefore be treated as central uncertainties.

Antioxidants and osmolytes present a second interpretive ambiguity: their accumulation may be protective, compensatory, or simply a marker of injury. Causal inference requires perturbation of the relevant gene or flux, paired with damage, recovery, and yield measurements. Heavy‐metal studies add another distinction: mechanisms that protect maize grain through root sequestration may conflict with strategies designed for phytoextraction (AbdElgawad et al. 2020; Lakhneko et al. 2024; Zamani et al. 2024).

## 9 Future Directions

Future work should use factorial, multi‐season field experiments with realistic drought‐salinity‐metal combinations and report grain yield, kernel set, harvest index, grain metal concentration, and genotype‐by‐environment effects alongside biochemical measurements. Mechanistic studies should combine organ‐ and cell‐resolved redox sensors with targeted perturbation of ABA‐SnRK2, ROS, transporter, osmolyte, and root‐architecture nodes. Breeding pipelines should validate edits and QTLs in elite backgrounds, quantify growth‐defense trade‐offs, and test stacked alleles against single‐gene controls. Standardized stress chemistries, preregistered sampling times, open phenotypic and omics data, and explicit separation of crop tolerance from phytoextraction would make studies more comparable and useful for deployment.

## 10 Conclusion

Maize acclimation to drought, salinity, and heavy metals depends on interacting ROS‐hormone signals, antioxidant recycling, osmolyte and carbon metabolism, ion transport, root architecture, and reproductive timing. The literature supports several maize‐specific regulators, but it also reveals opposing gene effects, strong organ and genotype dependence, and a persistent gap between seedling assays and field yield. Progress therefore requires causal, multi‐stress validation in elite germplasm, with grain productivity and food safety evaluated together.

## Funding

The authors have nothing to report.

## Ethics Statement

The authors have nothing to report.

## Consent

The authors have nothing to report.

## Conflicts of Interest

The authors declare no conflicts of interest.

## Data Availability

Data sharing not applicable to this article as no datasets were generated or analysed during the current study.
